# The Environmental Footprint Associated With the Mediterranean Diet, EAT-Lancet Diet, and the Sustainable Healthy Diet Index: A Population-Based Study

**DOI:** 10.3389/fnut.2022.870883

**Published:** 2022-05-19

**Authors:** Sigal Tepper, Meidad Kissinger, Kerem Avital, Danit Rivkah Shahar

**Affiliations:** ^1^Department of Nutritional Sciences, Tel-Hai College, Tel Hai, Israel; ^2^Department of Geography and Environmental Development, Ben Gurion University of the Negev, Beer-Sheva, Israel; ^3^The International Center of Health Innovation and Nutrition, Epidemiology, Biostatistics, and Community Health Sciences, School of Public Health, Faculty of Health Sciences, Ben-Gurion University of the Negev, Beer-Sheva, Israel

**Keywords:** sustainable diets, environmental footprints, EAT-Lancet, Mediterranean diet, sustainable food system

## Abstract

Providing a growing global population with healthy and sustainable diets is an immediate challenge. In the current study, estimates were obtained for the environmental footprints (land, water, and greenhouse gas (GHG) emissions) in association with the Mediterranean diet (MED) and the EAT-Lancet reference diet, which represents a healthy diet derived from sustainable food systems. We used a newly developed Sustainable Healthy Diet (SHED) index that was validated for the Israeli population by Tepper et al. in 2020.

**Methods:**

A group of 525 participants were recruited via social media, email, and phone. Demographic characteristics, quality of life, and answers to the SHED-index questionnaire were obtained. Dietary assessment was performed using the 116-item Food Frequency Questionnaire (FFQ), which was developed for the Israeli population. Adherence to the MED was calculated using a 9-point score. Adherence to the EAT-Lancet reference diet was assessed through the consumption of 14 food components. The environmental pressure of these dietary patterns was determined based on the “footprint family indicators,” which include land, water, and carbon footprints per unit of agricultural and food products. We assigned values for each food comprising the FFQ and calculated the environmental load for each dietary pattern. Statistical analyses were performed using the R package version 4.1.1 to compare environmental footprint values according to tertiles of the MED score, EAT-Lancet score, and SHED score.

**Results:**

The participants (*n* = 525) were 49% women, educated (82% had academic education), and physically active, and only 13% were smokers. The highest tertiles of adherence to the MED, adherence to the EAT-Lancet reference diet, and the SHED index were associated with the lowest GHG emissions and land use, as well as higher water use. Meat consumption contributed the most to land use, while dairy contributed the most to GHG emissions, and fruits contributed the most to water use.

**Conclusions:**

Our analysis reveals that animal protein is the highest contributor to GHG emissions and land use, while fruits and vegetables contribute the most to water consumption. Nevertheless, most of the fruits and vegetables are grown using treated wastewater, which reduces environmental pressure. Given these findings, we suggest that MED and EAT-Lancet dietary patterns should be included in national dietary guidelines.

## Introduction

The scientific interest in food consumption has evolved in recent decades, given its social and human health implications, direct and indirect pressure on domestic and global environmental systems, and contribution to the wellbeing of individuals and societies (1, 2). A sustainable food system ensures food security and nutrition for all while considering present and future economic, social, and environmental implications (2, 3). Nevertheless, providing a growing global population with healthy and sustainable diets is an immediate challenge (1, 3).

The Mediterranean diet (MED) is well known for its health benefits and has been identified for its environmental benefits as well (4). The diet is characterized by a high intake of plant-based foods; moderate to high intake of fish; moderate to low consumption of poultry, meat, and dairy; high intake of monounsaturated fatty acid (mainly from olive oil); and a moderate amount of wine (1–2 portions per day). As such, the MED's use of natural resources and environmental footprint have been revealed to be low (4, 5). The EAT-Lancet Commission has defined a reference “planetary health diet” based on both sustainability and health. The diet outlines a combination of food groups and ranges of food intake that could optimize human health and the environment (6). In a previous study, the Sustainable and Healthy Diet (SHED) index was developed and validated against both the MED and EAT-Lancet reference diet, which are both considered healthy and sustainable dietary regimens (7).

However, as dietary patterns are shaped by combined social and environmental factors, there is a need to examine not only recommended diets, but also those that are actually practiced, which is critical for advancing healthy and sustainable diets. Few recent studies have examined actual consumption patterns of individuals, households, and societies, their socio-demographic drivers, and their environmental and health implications (4, 5). One of these studies was performed among Italian adults and suggested that the adoption of healthy dietary patterns involves less use of natural resources and greenhouse gas (GHG) emissions (4). Similar results were shown among graduate students in Spain (5).

This paper joins this emerging direction of research in analyzing actual consumption patterns and exploring the gap between recommended diets and actually practiced ones, as well as the implications for the environmental impact of consumption of such diets. To this end, the study explores the differences in the environmental footprint (land, water, and GHGs) of different consumed diets based on local life cycle assessment (LCA) analysis, including unique aspects of the food system in Israel. For this purpose, we created an integrated database within the Food Frequency Questionnaire (FFQ), which includes environmental coefficients for environmental footprints. We analyzed our results with respect to the SHED index (7), the MED (4), and the EAT-Lancet dietary patterns (6) and evaluated the contribution of specific food groups to the environmental footprints using population-based cross-sectional data.

## Methods

### Study Population

Using social media, email, and phone calls, we recruited 525 men and women aged 20–66 years during 2018–2020. We approached predefined subpopulations such as vegans and vegetarians; people identifying as secular; rural and urban participants; and individuals with various environmental orientations. Culturally, the population of Israel consists of Jewish populations (the majority) and non-Jewish populations (Muslims, Christians, and Druze), which each have unique dietary consumption patterns. Therefore, the survey includes representation of both Arab and Jewish participants. Using data from the Central Bureau of Statistics in Israel, we aimed to obtain a representative sample of these subpopulations. Once achieving a representative sample for a specific sector, further respondents from this sector were excluded during phone interviews. Participants received the equivalent of 10 USD for completing the questionnaire.

### SHED Index

The SHED index is a newly developed, validated index that uses a 30-item questionnaire to assess healthy and sustainable individual diets. The score reflects the nutritional, environmental, and sociocultural aspects of sustainable diets (7). Responses to the items regarding sustainable and healthy eating are recorded on a Likert scale of 1–4. Items are ranked from “Almost never true” to “Almost always true” or from “Never” to “Most of the time.” Data on the consumption of beverages and pre-prepared meals are recorded on a scale of six frequencies from “Never” to “Daily.” Finally, participants are asked to rate the proportion of plant-based foods in their entire diet on a scale of 0–100%. The questionnaire includes information on demographics, lifestyle, location of food purchases, and frequency of food preparation.

### Dietary Assessment

Dietary assessment was performed using the 116-item FFQ, which was developed for the Israeli population. The development and validation process of this questionnaire are described in detail elsewhere (8). The questionnaire is updated annually using a database from the Israeli Ministry of Health (MOH). The MOH data are obtained from the changes and reformulations of food composition and consumption by the Israeli population. For the current study, an updated version from 2018 was used. Briefly, the FFQ includes 116 food items with nine frequency options ranging from “never or less than once monthly” to “six or more times daily.” The questionnaire is semi-quantitative, and a standard portion size is described for each food item. The portion-size estimates are based on information from the MOH. Participants are asked to report their average frequency of consumption during the past year. The questionnaire was self-administered electronically, thus ensuring completeness of the data as a participant could not complete the questionnaire if an item is not answered.

### Mediterranean-Diet Score

Adherence to the MED was calculated according to a 9-point score created by Trichopolou et al. (9). For each of the nine components except for alcohol, a value of 0 or 1 is assigned. The units of measurements are serving sizes, and the sex-specific medians of intake of the sample are used as cutoff points. One point is assigned for consumption that is above the median for each of the six protective components (fatty acid ratio, legumes, grains, fruits, vegetables, and fish), and one point is assigned if intake is below the median for the two non-protective components (dairy products and meat). For alcohol, one point is assigned for the mean consumption of 10–50 g/d for men and 5–25 g/d for women.

A score of 9 reflects maximum adherence, indicating that the participant meets all the characteristics of the MED. Based on a sensitivity analysis, we constructed three levels of adherence scores. Low adherence was defined as 0–3 points, medium adherence was 4–6 points, and high adherence was 7–9 points (10).

### EAT-Lancet Score

The EAT-Lancet score was calculated based on the calculation created by Kesse-Guyot et al. (11). This calculation is based on the components and cutoff of the EAT-Lancet diet that have been suggested by Willett et al. (6) regarding the consumption of the following 14 food components: whole grains, tubers and starchy vegetables, vegetables, fruits, dairy foods, beef/lamb/pork, poultry, eggs, fish, legumes, nuts, saturated fat, unsaturated fat, and added sugars. The score was computed using the following equation:


$$\begin{array}{l} Eat-Lacent\;score=\frac{\frac{100\times \{{\sum^{\text{​}}}_{component\;i=1}^{14}\;a_{i}\times (cut-off_{i}-\frac{consumption_{ij}*2500}{Energy\;consumption_{j}})\}}{cut-off_{i}}}{14} \end{array}$$

*i* refers to the 14 food groups, *j* is the individual participant in the study, *a*_*i*_ = 1 for a component to limit, and *a*_*i*_ = −1 for a component to promote.

### Demographics and Quality of Life

Socio-demographic and lifestyle data included age, sex, employment status, marital status, academic education, area of residence (degree of urbanization), religious identification, crowding (individuals per room), smoking status, level of physical activity, and weight status. Weight status was self-reported as underweight, normal weight, overweight, or obese. Most of these variables were classified as binary variables and reported as percentages. Health-Related Quality of Life (HRQOL) was measured according to the CDC's wellbeing tool and included—“unhealthy days,” indicating compromised physical or mental health in the last month, as well as self-rated general health.

### Data Collection of Dietary Consumption and Eating Patterns

Survey data were collected using a web application (Qualtrics software, version XM^©^, Provo, UT, USA. https://www.qualtrics.com). This application reduces missing data. Skipping questions is possible only with pre-definition and was allowed for only items that were decided in advance. The data were extracted in a CSV format and subjected to statistical analysis.

### Assigning Environmental Footprints Values

The environmental loads of the studied diets were based on the “footprint family indicators.” These indicators have been described as “a set of indicators, characterized by a consumption-based perspective, able to track human pressures on the surrounding environment, where pressure is defined as an appropriation of biological natural resources and CO_2_ uptake, emission of GHGs, and consumption and pollution of global freshwater resources” (12). It can be used to identify and assess environmental loads associated with a process, product, or system and allows for examination of potential bio-physical tradeoffs from proposed policy or other measures (12–14). The footprint family indicators of land, water, and carbon footprints per unit of agricultural and food products were analyzed, which required the integration of several kinds of data from different sources. In the following, we describe key data sources and present the calculation procedure for each biophysical indicator.

#### Land Footprint

The LF included the agricultural land area (*m*^2^) required for growing a unit (kg) of a commodity consumed in Israel. The analysis used data on dozens of crops and processed products from FAOstat (13) to allocate a country's food supply. The allocation procedure first converts processed products and livestock items to primary crop equivalents. Data on commodities grown locally were obtained from FAOstat data, and a bilateral trade matrix was constructed for each crop imported over the last decade. A full description of the data and procedures is available from previous studies (14, 15). Concordance tables and conversion factors used in this analysis are provided as supplementary information.

#### Water Footprint

The analysis of the water demand for each of the food items included in the research was performed using a database on virtual water (16). The analysis was done using the trade matrix described above to identify domestic and imported food items. The water footprint presented in this research focused on blue water (i.e., irrigation water in cubic meters per ton) in different regions of the world related to the supply of food for consumption in Israel.

#### Carbon Footprint

The CF of each commodity was calculated by incorporating carbon dioxide, nitrous oxide, and methane emissions along the commodity chain for one ton of food consumed in Israel. The results are presented in CO_2_ equivalents (CO2e) using factors of 1 kg CO_2_/kg CO_2_, 310 kg N_2_O/kg CO_2_, and 21 kg CH_4_/kg CO_2_ (17). The related emissions integrated two types of data sources. The first followed detailed carbon-footprint review studies, including one by Heller et al. (18), and the second relied on a series of studies based on life-cycle assessments (LCAs) in Israel [e.g., for beef (19), poultry (16), dairy (20), peppers (21), and dates (22)]. In addition, this study also used relevant data for other domestically grown commodities that are now under review and preparation (tomatoes, grapes, avocadoes, and others).

The environmental load values were integrated together into the FFQ to calculate LF, WF, and CF for the questionnaire. Since the survey was designed for nutritional assessment, several nutritionally comparable food items were grouped in the FFQ, along with recipes that were added. In order to assign environmental load values, these groupings and recipes were disaggregated into the original list of 507 food items that originate from the MABAT survey (23). As a result of the integration between the environmental load and dietary intake and quality, each line in the FFQ includes macro and micronutrients and the associated environmental loads of these items.

The total environmental loads for each participant's diet were assessed using the weights of each food item and frequency of consumption. We summed the values of all food items and obtained the impact on the water, land, and GHG emissions of the daily diet of each participant. Although we obtained information for most food items, for a few of them, we did not have available data on their environmental sustainability characteristics. In those cases, we assigned the item the value of the most similar item. However, we did not have enough data for soft drinks, alcoholic drinks, and fish. In addition, data about the LCA for ultra-processed foods were lacking, so they were not included in the current analyses.

### Statistical Analysis

All statistical analyses were performed using the R statistical environment (R version 4.1.1), along with a number of libraries that extend the capabilities of the core version of the program. The most significant R libraries used in the analyses were ggplot2, ggpubr, and gtsummary. Dietary intake of food groups and environmental footprints values were represented with the means and standard deviations. Categorical variables were tested using Pearson's Chi-squared test or Fisher's exact test. Fisher was used in cases where there were <5 subjects in one of the groups. Continuous variables were tested with Kruskal-Wallis tests. We classified each participant based on tertiles of the SHED score, MED score, and EAT-Lancet score (4, 6, 7). Kruskal-Wallis tests were used to assess the differences between the tertiles of each score in regard to GHG emissions, land use, and water use.

## Results

The sample included 525 young adult participants aged 20–66 years. The study participants were educated (82% had an academic education) and physically active, and only 13% were smokers. Higher SHED scores were associated with older age, women, higher education, non-smokers, normal weight, flexitarians, and vegetarian/vegans (Table 1). The same trends were observed for MED and EAT-Lancet scores (not shown).

**Table 1:** Characteristics of the study population according to tertiles of the Sustainable and Healthy Eating (SHED) Index score.

	**SHED tertiles**				
**Characteristic**	**Overall**, ***N*** **=** **525^a^**	**Low^a^**	**Medium^a^**	**High^a^**	***p*** **-value^b^**
Age (years)	32 (10)	30 (8)	32 (10)	35 (10)	<0.001
Gender (women)	259 (49%)	54 (31%)	86 (49%)	119 (68%)	<0.001
Married	259 (49%)	78 (45%)	89 (51%)	92 (52%)	0.4
Employed	396 (76%)	133 (77%)	126 (72%)	137 (78%)	0.4
People per room	0.85 (0.34)	0.88 (0.34)	0.86 (0.37)	0.82 (0.30)	0.2
Education (academic)	432 (82%)	129 (75%)	150 (86%)	153 (87%)	0.004
Smoking	66 (13%)	30 (17%)	21 (12%)	15 (8.5%)	0.043
Physical activity (minutes/week)	161 (155)	127 (152)	169 (155)	186 (152)	<0.001
Self- rated poor health^c^	18 (3.4%)	7 (4.0%)	7 (4.0%)	4 (2.3%)	0.6
Sum of unhealthy days^c^	4.5 (6.0)	4.6 (6.2)	4.7 (6.2)	4.3 (5.6)	0.8
Weight status^d^					0.059
Normal	332 (63%)	98 (57%)	107 (61%)	127 (72%)	
Obese	42 (8.0%)	14 (8.1%)	17 (9.7%)	11 (6.2%)	
Overweight	146 (28%)	58 (34%)	51 (29%)	37 (21%)	
Eating pattern					<0.001
Flexitarian	78 (15%)	9 (5.2%)	22 (12%)	47 (27%)	
High animal-based food	77 (15%)	39 (23%)	21 (12%)	17 (9.7%)	
Omnivore	312 (60%)	121 (70%)	124 (70%)	67 (38%)	
Vegetarian/vegan	56 (11%)	3 (1.7%)	9 (5.1%)	44 (25%)	

a *Mean (SD); n (%). SHED tertile values: tertile 1: SHED <17, tertile 2: 17 ≤SHED <27.9, tertile 3: SHED ≥27.9*.

b *Kruskal-Wallis rank sum test; Pearson's Chi-squared test; Fisher's exact test*.

c *Summary from Health-Related Quality of Life (HRQOL) questions—unhealthy days with compromised physical or mental health in the last month and self-rated general health*.

d *Self-reported weight status*.

**Figure 1:**
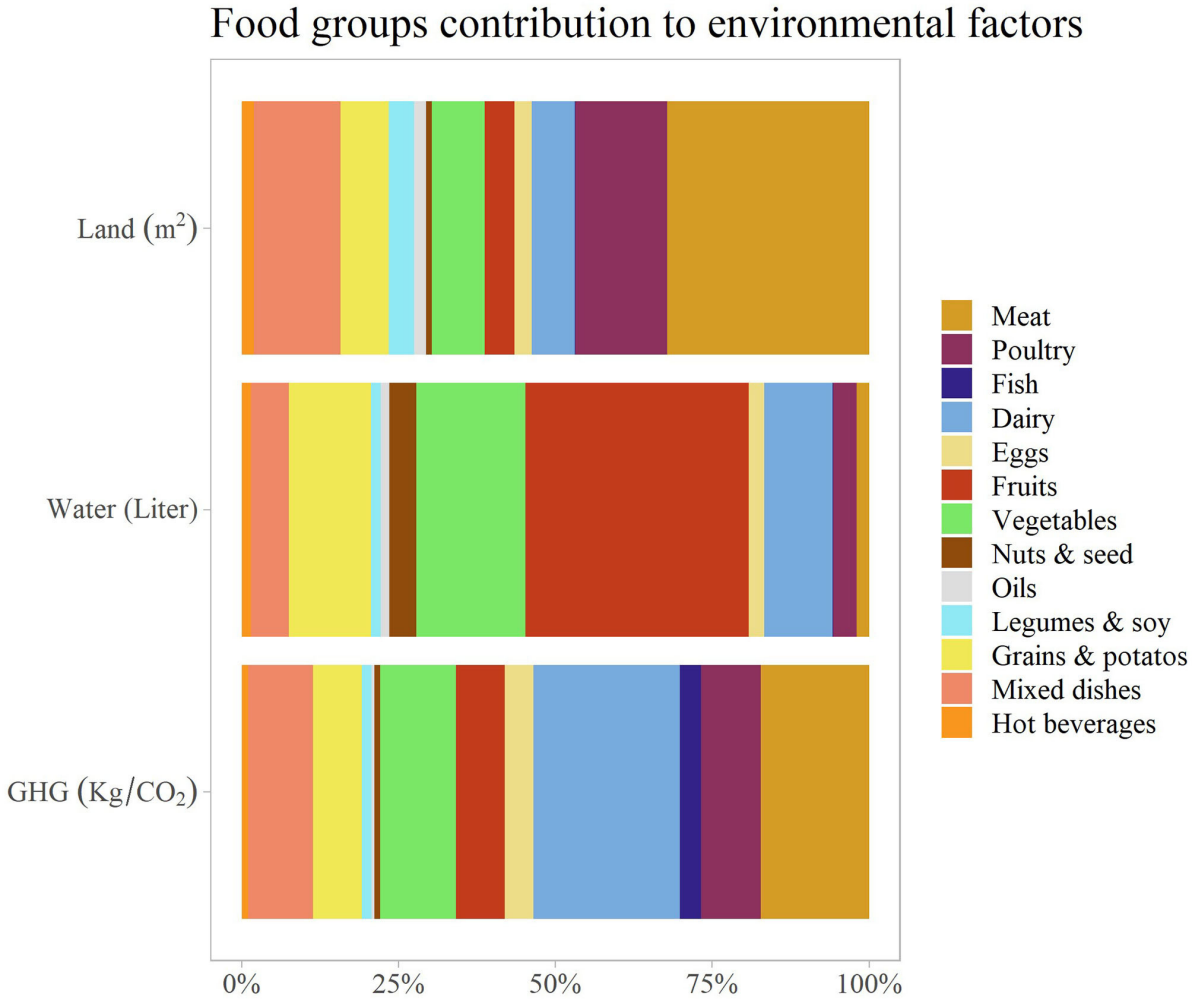
Food groups' relative contributions to land, water, and GHGs footprints.

The mean footprint use of the total sample was 5.7 ± 3.8 m^2^ for land, 422 ± 229 liters for water, and 2.84 ± 1.32 kg/CO_2_ for GHGs. The food groups' contributions to the different environmental footprints differ across factors (Figure 1). The main contributor to water use was fruits (40%), followed by vegetables (12%) and dairy (11%). The main contributor to land use was meat (30% for beef and 14% for poultry), and the main contributor to GHG emissions was dairy products (26%), followed by meat (14%) and vegetables (14%).

**Figure 2:**
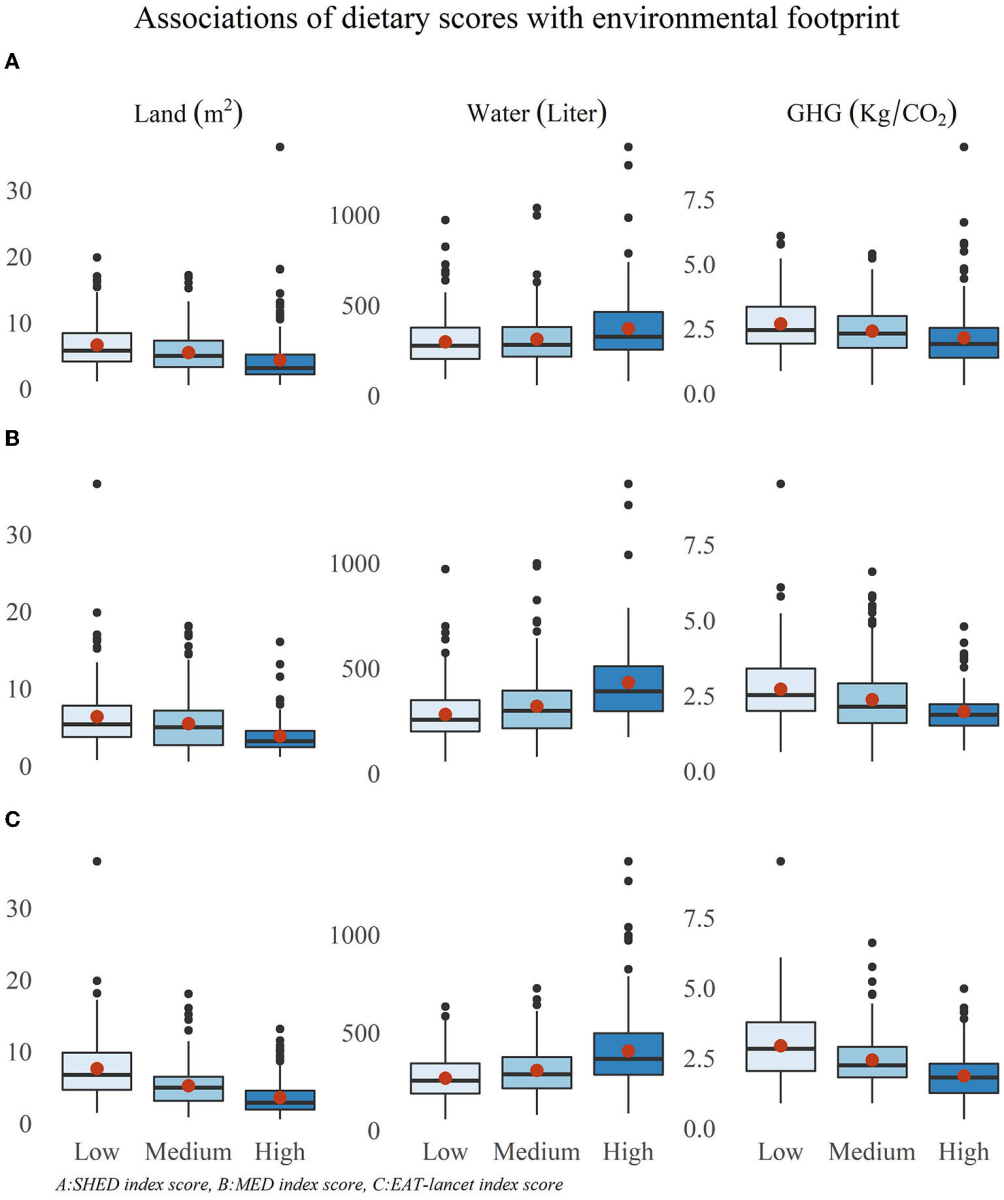
Environmental footprint of different dietary patterns. *All *P* < 0.0001 (Kruskal-Wallis Test).

**Figure 3:**
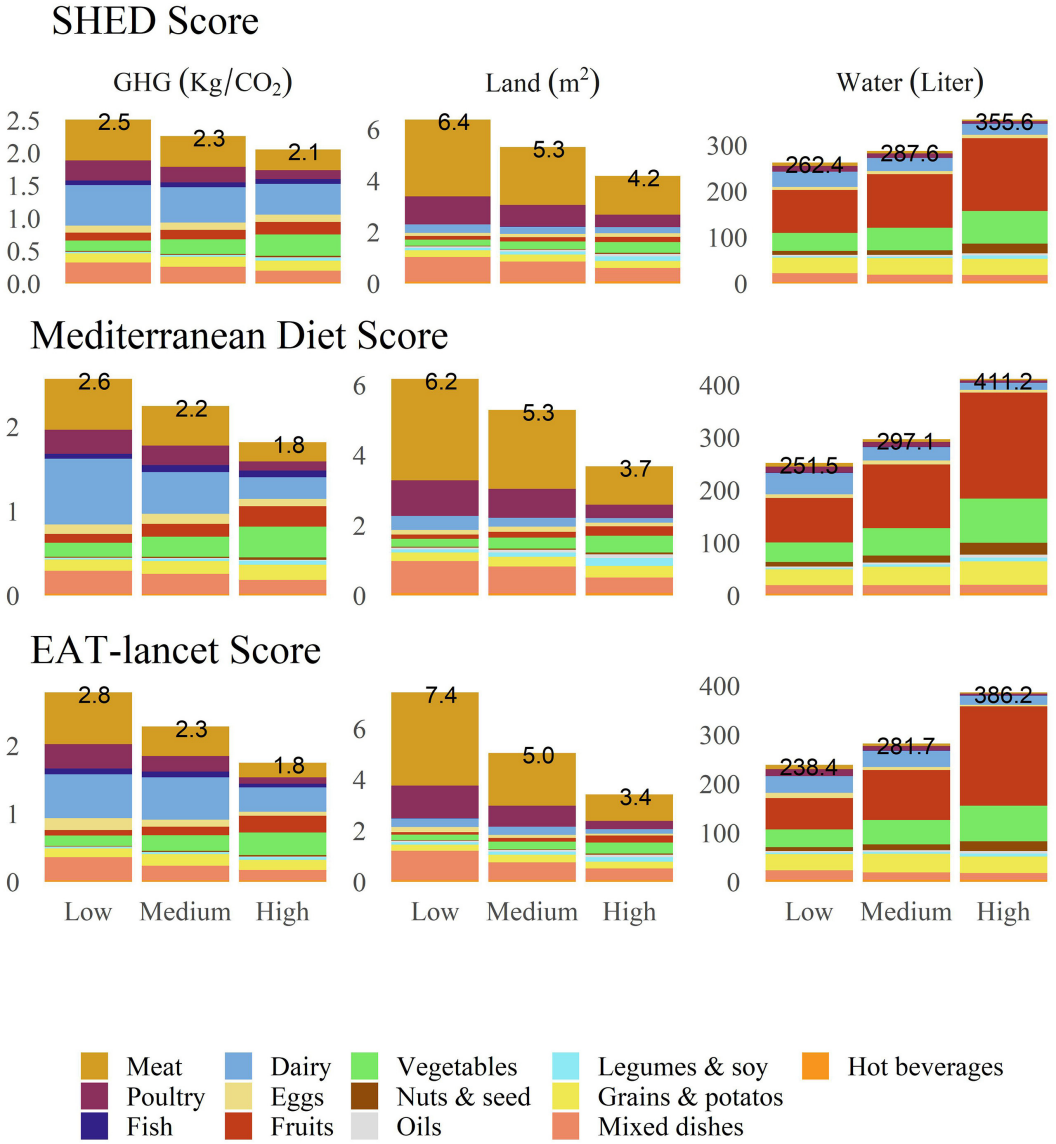
Food groups' contribution to environmental footprints for different dietary patterns. *All *P* < 0.0001 (Kruskal-Wallis Test).

Next, the environmental factors were calculated according to adherence to MED and EAT-Lancet dietary patterns and according to tertiles of the SHED scores (Figure 2). Higher adherence to MED was associated with lower land use (high vs. low adherence: 4.07 ± 2.63 vs. 6.6 ± 4.3 m^2^, *p* < 0.001), GHG emissions (high vs. low adherence: 2.32 ± 0.9 vs. 3.21 ± 1.32 kg/CO_2_, *p* < 0.001), and higher water use (high vs. low adherence: 560 ± 281 vs. 360 ± 203 liters, *p* < 0.001). The same trend was observed for EAT-Lancet adherence and SHED scores (all *p* < 0.001). Figure 3 shows the mean contribution to the environmental footprints by food groups for different dietary patterns. At each adherence level of the dietary scores, there was a similar ranking of the food groups to that in Figure 1. The main contributor to GHG emissions was dairy products, followed by meat. The main contributor to land use was meat, and the main contributor to water use was fruit intake at each adherence level.

To further explore the relationship between the food groups and the environmental coefficients, we calculated the average consumption of the food-group items for different tertiles of GHG emissions, land use, and water use. Table 2 shows the mean daily intake of the different food groups by tertiles of environmental footprints. The consumption of plant food groups (fruits, vegetables, nuts/seeds, legumes/soy, and grains/potatoes) did not differ for different land tertiles with the exception of plant oils. The consumption of animal-based food increased for different land tertiles, demonstrating high use of land resources with increased consumption.

**Table 2:** Mean daily intake of food groups by tertiles of environmental footprints.

	**Land tertiles**				**GHG tertiles**				**Water tertiles**				
**Food Group**	**Low^a^**	**Medium^a^**	**High^a^**	***p*-value^b^**	**Low^a^**	**Medium^a^**	**High^a^**	***p*-value^b^**	**Low^a^**	**Medium^a^**	**High^a^**	***p*-value^b^**	**Overall, *N* = 525^a^**
Meat	5 ± 6	21 ± 12	61 ± 39	<0.001	9 ± 11	21 ± 16	57 ± 43	<0.001	22 ± 24	28 ± 28	37 ± 44	0.021	29 ± 34
Poultry	32 ± 33	86 ± 59	132 ± 80	<0.001	41 ± 46	78 ± 56	131 ± 82	<0.001	71 ± 56	86 ± 82	94 ± 77	0.062	83 ± 73
Fish	16 ± 20	26 ± 31	27 ± 23	<0.001	15 ± 18	23 ± 20	31 ± 33	<0.001	18 ± 17	24 ± 27	27 ± 30	0.032	23 ± 25
Dairy	323 ± 335	389 ± 339	493 ± 454	<0.001	220 ± 216	386 ± 305	599 ± 485	<0.001	345 ± 300	393 ± 365	468 ± 465	0.086	402 ± 385
Eggs	22 ± 22	34 ± 42	55 ± 76	<0.001	16 ± 15	35 ± 36	60 ± 78	<0.001	24 ± 27	37 ± 44	50 ± 75	<0.001	37 ± 53
Fruits	348 ± 405	323 ± 259	362 ± 349	0.2	273 ± 257	373 ± 381	387 ± 367	<0.001	128 ± 76	274 ± 125	631 ± 445	<0.001	344 ± 343
Vegetables	547 ± 351	558 ± 402	643 ± 556	0.5	471 ± 303	595 ± 408	681 ± 565	<0.001	345 ± 219	555 ± 294	848 ± 580	<0.001	582 ± 446
Nuts seed	11 ± 12	12 ± 15	10 ± 11	0.8	9 ± 11	13 ± 16	11 ± 11	0.3	6 ± 6	10 ± 11	17 ± 17	<0.001	11 ± 13
Oils	4.2 ± 3.6	5.4 ± 5.5	5.8 ± 5.1	0.011	4.2 ± 4.4	5.4 ± 4.8	5.9 ± 5.2	<0.001	3.5 ± 3.2	5.1 ± 4.2	6.9 ± 6.1	<0.001	5.2 ± 4.8
Legumes/soy	84 ± 109	65 ± 62	60 ± 64	0.3	81 ± 108	66 ± 74	62 ± 53	0.5	47 ± 55	66 ± 59	96 ± 111	<0.001	70 ± 82
Grains potatoes	202 ± 131	214 ± 132	230 ± 133	0.074	181 ± 109	219 ± 134	245 ± 144	<0.001	165 ± 83	220 ± 130	261 ± 155	<0.001	215 ± 132
Mixed dishes	104 ± 73	149 ± 86	220 ± 125	<0.001	112 ± 73	149 ± 85	214 ± 131	<0.001	118 ± 78	157 ± 97	199 ± 128	<0.001	158 ± 108
Hot beverages	485 ± 373	499 ± 455	582 ± 489	0.2	458 ± 378	485 ± 423	622 ± 504	0.016	420 ± 364	551 ± 426	595 ± 511	0.004	522 ± 443
Total consumption	3,418 ± 1,084	3,720 ± 1,023	4,283 ± 1,219	<0.001	3,134 ± 943	3,760 ± 927	4,527 ± 1,170	<0.001	2,937 ± 868	3,762 ± 720	4,722 ± 1,103	<0.001	3,807 ± 1,166

a *Mean consumption g/day ± SD*.

b *Kruskal-Wallis rank sum test*.

The meat intake was 12 times higher in the upper tertile of land use than in the lowest tertile, while poultry intake was 4 times higher, and egg intake was 2.5 times higher. Regarding GHG emissions, only the consumption of nuts/seeds and legumes/soy did not differ between tertiles. In the case of water, only poultry and dairy did not differ between tertiles. These results indicate a narrow range of consumption and smaller influence on water use and GHG emissions.

## Discussion

The need to assess both human health and environmental footprint of diets has been widely acknowledged. Nevertheless, most studies have focused on theoretical models of dietary guidelines and their potential contributions to sustainability and human health rather than actual consumption (3). The analysis presented in this paper joins a limited number of studies that have identified this gap (4, 5, 24). By analyzing data from a diverse sample of 525 people in Israel, we evaluated real consumption data in regard to adherence to healthy and sustainable diets. These findings are the integrative product of both the studied population's consumption habits and the environmental factors of each studied commodity.

Our findings indicate that the main contributors to water use were fruits, vegetables, and dairy. The main contributors to land use were meat and poultry, and the main contributor to GHG emissions was dairy products. We found that the highest tertiles of adherence to the MED and the EAT-Lancet reference diet (6, 9) were associated with the lowest GHG emissions and land use. On the other hand, the highest tertiles of adherence to the MED and EAT-Lancet were associated with higher water use. To expand our view on sustainability beyond the environmental footprint, adjoining sociocultural, economic, and health aspects we used the SHED index (7). The need to develop methods to include all 4 dimensions of sustainability of the diet (in their case MED) were acknowledged in a recent review by Portugal et al. (24). The results of the SHED index analysis were similar to those obtained for the MED and EAT-Lancet dietary pattern.

The health value of the MED is well established. During the last 20 years MED was shown to benefit health and function, reducing mortality rates (9, 10, 25). The EAT-Lancet as a theoretical dietary pattern targets both health and the environment using evidence based data, indicating that healthy and sustainable diet is achievable (11, 26). In a review by Aleksandrowicz et al. (1), the authors calculated the potential shift in footprint values associated with different dietary patterns. They conclude that shifting from Western diets to more environmentally sustainable dietary patterns can reduce above 70% of GHG emissions and land use, and 50% of water use.

Our findings parallel those of prior studies. In studies from Italy and Spain that used real consumed diet, high adherence to MED pattern was associated with lower GHG emissions and land use (4, 5). The contribution of animal products (meat, poultry, dairy, egg, and fish) constituted the greatest contributor to GHG emissions (19, 21, 22, 27). Higher adherence to the MED or the EAT-Lancet recommended diet was associated with lower GHG emissions in other studies In Spain (the SUN cohort) better adherence to the Spanish MED was associated with decreased environmental pressures in all assessed dimensions including GHG, land and water (5). Likewise, a cross-sectional study among Italian adults showed that omnivorous dietary choices or low adherence to the MED correlated with higher GHG emissions, land, and water use (4, 25). Results in the same direction were found in a non-MED country. Data from the European Prospective Investigation into Cancer and Nutrition—Netherlands (EPIC-NL) (28) cohort showed that the WHO and Dutch dietary guidelines lower the risk of all-cause mortality and moderately lower the environmental impact, while the DASH diet (Dietary Approaches to Stop Hypertension diet), despite leading to similar health outcomes, was associated with higher GHG emissions due to high dairy product consumption in the Netherlands (29). Another study conducted on Italian adults showed that omnivorous dietary choices generated worse carbon, water, and ecological footprints than other plant-based diets, while no differences were found for the environmental impacts of ovo-lacto-vegetarians and vegans (28). Unlike the above studies, in our findings water footprint was higher in the third tertiles of MED, EAT-Lancet and SHED and was connected tofruit intake.

This unique aspect revealed in our analysis needs further discussion. Since fruits and vegetables are more dependent on irrigation than animal-based foods, reducing animal-based foods and increasing plant-based foods do not always correspond with lower water use, as shown by Harris et al. (30). Plant-based foods were major contributors to dietary blue-water footprints. Grosso et al. (4) also found that higher adherence to the MED was not linearly associated with lower water consumption. Higher fruit consumption was also associated with higher water footprints in the United States, particularly the blue-water footprint (17).

However, theoretical dietary models show different results indicating that shifting to a more plant-based diet would reduce the water footprint (31). Other studies also demonstrate that the contribution of fruits and vegetables does not exceed the meat contribution for both blue and green water (32). Indeed, we found that sustainable diets like MED or the EAT-Lancet reference diet are characterized by higher water consumption, but several considerations need to be taken into account. One is that most fruits and vegetables consumed in Israel are grown locally. It follows that given the climatic conditions, most are irrigated, so they would have relatively high rates of blue-water footprints.

Nevertheless, it is important to note that not all blue water is the same as most of the fruit-related water footprint relies on treated wastewater, which reduces environmental pressure (33). Other solutions such as the use of desalinated water and efficient and cost-effective irrigation techniques already exist in Israel, but our findings emphasize the importance of further development of water management, including advanced technologies, reducing water losses, and improving data quality and monitoring for water–food system linkages (32, 33).

Management of the local food systems can also reduce water consumption. There are substantial differences of 2–10-fold in water consumption between different fruit crops (34). Prioritizing certain types of crops that are less burdensome in terms of water requirements while considering their health benefits could be another future direction to increase the health and sustainability of food systems.

As for dairy intake in Israel, the mean intake in our study is 402 ± 385 g per day. This value is higher than the estimated intake in Europe and North America, where the average daily intake is 364.8 g per day according to the PURE study (35). Since most dairy products consumed in Israel are domestically produced, and the production system is based on non-grazing cows, production can occur in a relatively small area (19, 27). In addition, the productivity of Israeli dairy cows is very high, which reduces the footprint per unit of milk (20). Nevertheless, the high demand for dairy products identified in our analysis led to high rates of dairy-related footprints.

According to the EAT-Lancet commission (6) and in accord with the national dietary recommendations, the requirement for different food groups is calculated based on healthy dietary intake within global boundaries. For example, the reference intake is 29 g per day for poultry, 300 g per day for vegetables, 200 g per day for fruits, and 250 g per day for dairy. In our data (Table 2), the actual consumption in the lowest tetiles of land GHG and water was nearly similar to the EAT-Lancet recommended diet. The main difference was fruit consumption. Thus, a shift, toward less animal based and more plant-based diets, is beneficial for both health and the environment. The Isocaloric Substitution of Plant-Based and Animal-Based Protein was related with Aging-Related Health Outcomes (36). A recent paper by Eisen and Brown, (37) show that, following a phaseout of livestock production will independently provide persistent drops in atmospheric methane and nitrous oxide levels, and slower carbon dioxide accumulation. This reduction through the end of the century, have the same cumulative effect on the warming potential of the atmosphere as a 25 gigaton per year reduction in anthropogenic CO2 emissions. This level of reduction will provide half of the net emission reductions necessary to limit warming to 2C.

Based on our data, which originate from the FFQ results of 525 participants, it seems that there is no conflict between a healthy and sustainable diet, but there is a need to adjust and optimize dietary patterns in light of recommendations for various populations with different dietary needs. For example, Israel is characterized with mixed Jewish and non-Jewish population, locals and new and established immigrants; and a significant young population alongside a growing share of elderly population. It is important to note that the EAT-Lancet reference diet stems from a theoretical model for a healthy and sustainable diet, whereas our data represent actual dietary patterns of the Israeli population. The results may be a proof of concept that the EAT-Lancet reference diet is indeed feasible.

### Strength and Weaknesses

One strength of this study is its ability to assign environmental-footprint values to the FFQ lines. The Israeli FFQ was created based on 24-h recall information that was collected in the Israeli National Health and Nutrition Survey (MABAT) (23). The results of MABAT were available to our group, so we could assign Environmental Footprint values to most of the 570 food items that were on the basic list for the FFQ. The final 116 lines of the FFQ were extracted from the 570 food items. In many cases, when the FFQ is used, the data behind the questionnaire are not available to the researchers. We believe that the use of this basic method results in a more accurate long-term assessment of EF exposure of our participants (8, 38).

Our footprint analysis was based on local supply coefficients. It follows that each analyzed food commodity footprint is considered in terms of whether it was supplied from local sources or imported from several other parts of the world. The footprint was then calculated to reflect the amount of land and water related to the supply from each source (14, 27, 32, 39, 40).

Our study also has several limitations that need to be addressed. One is the use of a convenience sample that was restricted to people who have access to web-based platforms. However, we made an effort to recruit a representative sample including all sectors in Israel. Our sample consists of a high number of educated participants who practice a healthy lifestyle, which may partially limit the generalizability of the results to the general population. While the footprint figures included detailed place-based data and calculations, for some commodities, we had to make some assumptions or exclude some footprint categories. For example, in the case of fish-related footprints, we included only global averages of GHG data and not the other footprint coefficients.

## Conclusion

Our analysis of consumed diets revealed that animal protein is associated with the highest GHG emissions and land use, while fruits and vegetables were associated with the highest water consumption. Nevertheless, most of them are grown using treated wastewater, which reduces environmental pressure. The differences in water consumption for different fruit crops support the need to prioritize certain types of crops, which should be less burdensome in terms of water requirements while considering their health benefits. Given these findings, we suggest that adherence to MED and EAT-Lancet dietary patterns should be included in national dietary guidelines and encouraged for consumption by all. Furthermore, our data could be used as a database to create healthy and sustainable diet recommendations while adjusting for nutritional needs and health status, as well as maintaining diversity within dietary patterns.

## Data Availability Statement

The raw data supporting the conclusions of this article will be made available by the authors, without undue reservation.

## Ethics Statement

The studies involving human participants were reviewed and approved by the Ethics Committee of Tel-Hai College (#09/2017-2). The patients/participants provided their written informed consent to participate in this study.

## Author Contributions

ST planned and conducted the research and wrote the first draft of the manuscript. MK calculated the environmental footprints and co-authored the manuscript. DS created the combined database and co-authored the manuscript. KA analyzed the data and co-authored it. All authors contributed to the article and approved the submitted version.

## Funding

This study was supported by Ben-Gurion University's internal fund for nutritional research, MIGAL - Galilee Research Institute grant, and Tel-Hai College research fund.

## Conflict of Interest

The authors declare that the research was conducted in the absence of any commercial or financial relationships that could be construed as a potential conflict of interest.

## Publisher's Note

All claims expressed in this article are solely those of the authors and do not necessarily represent those of their affiliated organizations, or those of the publisher, the editors and the reviewers. Any product that may be evaluated in this article, or claim that may be made by its manufacturer, is not guaranteed or endorsed by the publisher.
